# Comparison of Sleeve Gastrectomy vs Roux-en-Y Gastric Bypass: A Randomized Clinical Trial

**DOI:** 10.1001/jamanetworkopen.2023.53141

**Published:** 2024-01-30

**Authors:** Suzanne Hedberg, Anders Thorell, Johanna Österberg, Markku Peltonen, Ellen Andersson, Erik Näslund, Jens Kristoffer Hertel, Marius Svanevik, Erik Stenberg, Martin Neovius, Ingmar Näslund, Mikael Wirén, Johan Ottosson, Torsten Olbers

**Affiliations:** 1 Department of Surgery, Department of Clinical Sciences, The Sahlgrenska Academy, University of Gothenburg, Gothenburg, Sweden; 2 Department of Surgery (Östra Sjukhuset), Sahlgrenska University Hospital, Gothenburg, Sweden; 3 Department of Clinical Sciences, Danderyd Hospital, Karolinska Institutet, Stockholm, Sweden; 4 Department of Surgery, Ersta Hospital, Stockholm, Sweden; 5 Department of Surgery, Mora Hospital, Mora, Sweden; 6 Department of Clinical Sciences, Intervention and Technology (CLINTEC), Karolinska Institutet, Stockholm, Sweden; 7 Finnish Institute for Health and Welfare, Helsinki, Finland; 8 Department of Surgery, Vrinnevi Hospital, Norrköping, Sweden; 9 Department of Biomedical and Clinical Sciences, Linköping University, Linköping, Sweden; 10 Department of Endocrinology, Obesity, and Nutrition, Vestfold Hospital Trust, Tønsberg, Norway; 11 Department of Surgery, Vestfold Hospital Trust, Tønsberg, Norway; 12 Department of Surgery, Faculty of Medicine and Health, Örebro University, Örebro, Sweden; 13 Division of Clinical Epidemiology, Department of Medicine (Solna), Karolinska Institutet, Stockholm, Sweden; 14 Wallenberg Centre for Molecular Medicine, Department of Biomedical and Clinical Sciences, Linköping University, Linköping, Sweden

## Abstract

**Importance:**

Laparoscopic sleeve gastrectomy (SG) and laparoscopic Roux-en-Y gastric bypass (RYGB) are widely used bariatric procedures for which comparative efficacy and safety remain unclear.

**Objective:**

To compare perioperative outcomes in SG and RYGB.

**Design, Setting, and Participants:**

In this registry-based, multicenter randomized clinical trial (Bypass Equipoise Sleeve Trial), baseline and perioperative data for patients undergoing bariatric surgery from October 6, 2015, to March 31, 2022, were analyzed. Patients were from university, regional, county, and private hospitals in Sweden (n = 20) and Norway (n = 3). Adults (aged ≥18 years) eligible for bariatric surgery with body mass indexes (BMIs; calculated as weight in kilograms divided by height in meters squared) of 35 to 50 were studied.

**Interventions:**

Laparoscopic SG or RYGB.

**Main Outcomes and Measures:**

Perioperative complications were analyzed as all adverse events and serious adverse events (Clavien-Dindo grade >IIIb). Ninety-day mortality was also assessed.

**Results:**

A total of 1735 of 14 182 eligible patients (12%; 1282 [73.9%] female; mean (SD) age, 42.9 [11.1] years; mean [SD] BMI, 40.8 [3.7]) were included in the study. Patients were randomized and underwent SG (n = 878) or RYGB (n = 857). The mean (SD) operating time was shorter in those undergoing SG vs RYGB (47 [18] vs 68 [25] minutes; *P* < .001). The median (IQR) postoperative hospital stay was 1 (1-1) day in both groups. The 30-day readmission rate was 3.1% after SG and 4.0% after RYGB (*P* = .33). There was no 90-day mortality. The 30-day incidence of any adverse event was 40 (4.6%) and 54 (6.3%) in the SG and RYGB groups, respectively (odds ratio, 0.71; 95% CI, 0.47-1.08; *P* = .11). Corresponding figures for serious adverse events were 15 (1.7%) for the SG group and 23 (2.7%) for the RYGB group (odds ratio, 0.63; 95% CI, 0.33-1.22; *P* = .19).

**Conclusions and Relevance:**

This randomized clinical trial of 1735 patients undergoing primary bariatric surgery found that both SG and RYGB were performed with a low perioperative risk without clinically significant differences between groups.

**Trial Registration:**

ClinicalTrials.gov Identifier: NCT02767505

## Key Points

**Question:**

Do perioperative outcomes differ between laparoscopic sleeve gastrectomy and laparoscopic Roux-en-Y gastric bypass?

**Findings:**

In this large, Swedish-Norwegian, multicenter randomized clinical trial, perioperative complication rates were low and not statistically different between groups. Operating time was shorter for those who underwent a sleeve gastrectomy.

**Meaning:**

Both sleeve gastrectomy and Roux-en-Y gastric bypass can be performed with a similar and low perioperative risk; therefore, among adult patients with obesity undergoing primary bariatric surgery, the perioperative risk should not be a main deciding factor in the choice of method.

## Teaser

This randomized clinical trial compares perioperative outcomes among adults undergoing sleeve gastrectomy and laparoscopic Roux-en-Y gastric bypass.

## Introduction

Obesity is a major health concern, with a worldwide prevalence in adults of 13% and an estimated 4 million deaths annually linked to overweight and obesity.^1^ Metabolic and bariatric surgery is a well-documented treatment of severe obesity, leading to superior long-term weight loss,^2,3^ improvement or resolution of obesity-related morbidity,^4,5,6,7^ and reduced mortality^8,9^ compared with nonsurgical management. Approximately 600 000 primary surgical bariatric procedures were performed worldwide in 2018, with sleeve gastrectomy (SG) and Roux-en-Y gastric bypass (RYGB) the most prevalent.^10^ The comparative efficacy and safety of SG vs RYGB remain unclear.

Sleeve gastrectomy was recognized as a stand-alone procedure by the American Society for Metabolic and Bariatric Surgery in 2012^11^ and rapidly became the most popular bariatric technique in the US. Data from nonrandomized short- and intermediate-term studies include lower complication rates after SG compared with RYGB^12,13,14^ and suggest comparable outcomes between SG and RYGB in terms of weight loss, resolution of type 2 diabetes,^15^ hypertension,^16^ and hyperlipidemia.^17^ Important issues for comparison include long-term weight outcome^14^ and risk of gastroesophageal reflux disease.^18,19^

In 2012, 93% of all primary bariatric surgical procedures in Sweden were RYGB,^20^ with a gradual transition to an even frequency of SG and RYGB from 2017 and thereafter.^21^ Roux-en-Y gastric bypass has a long track record, and available observational data demonstrate sustained weight loss and improvement in obesity-related comorbidities.^2,22^ Challenges regarding the RYGB procedure include risk of small bowel obstruction,^23^ abdominal pain,^24,25^ postbariatric hypoglycemia,^26,27^ nutritional deficiencies,^28^ alcohol use disorder,^29,30^ and self-harm.^31^

Data from 2 well-designed, limited-sized, European randomized clinical trials comparing SG and RYGB demonstrate only minor differences regarding weight loss and resolution of comorbidities over 5 years.^32,33^ In addition, the risk of serious adverse events in the short- and intermediate-term follow-up did not differ between the groups. Randomized clinical trials of patients with type 2 diabetes show superior improvements in glucose control and diabetes remission after RYGB compared with SG.^4,34^

Lack of conclusive data from randomized studies, together with a rapid increase in the use of SG in both Sweden and Norway, prompted a large-scale randomized clinical trial comparing SG with RYGB regarding weight loss and risk of adverse events. We present baseline and surgical data as well as perioperative outcomes for SG and RYGB in a large, Swedish-Norwegian randomized clinical trial.

## Methods

The Bypass Equipoise Sleeve Trial (BEST) design, surgical procedures, and statistical analysis plan have been described previously.^35^ In brief, BEST is a Swedish-Norwegian, registry-based, open-label, pragmatic multicenter randomized clinical trial comparing midterm (5-year) outcomes of SG and RYGB.^35^ We consider this a pragmatic trial in that not all details were minutely controlled while controlling for surgical technique and a number of other mentioned variables; otherwise, the trial was performed in real-life, routine practice. Coprimary end points at 5 years are efficacy (weight loss) (>95% power) and safety (incidence of predefined adverse events) (80% power). In this article, we report perioperative outcome data (0-30 days) and 90-day mortality, using a per-protocol analysis, with participants analyzed according to the procedure they received. The statistical analysis plan and trial protocol for this perioperative analysis, including updated power calculation for the primary outcomes, are given in Supplement 1. This trial was conducted in accordance with the ethical standards of the Declaration of Helsinki^36^ and approved by the Regional Ethical Review Board in Gothenburg, Sweden, and the Regional Committee for Medical and Health Research Ethics in Norway. Written informed consent was provided by all participants. This study followed the Consolidated Standards of Reporting Trials (CONSORT) reporting guideline.^37^

Trial data (baseline characteristics, surgical data, and follow-up data) were recorded in the Scandinavian Obesity Surgery Registry (SOReg)—a quality registry for bariatric surgery in Sweden and Norway; thus, SOReg also served as the trial’s database. SOReg covers more than 98% of all bariatric surgical procedures performed in Sweden and 88% in Norway. The registry has a high validity of registered data,^38,39^ and the variables in the registry are identical in Sweden and Norway.

### Patients

Patients 18 years or older with a body mass index (BMI) of 35 to 50 (calculated as weight in kilograms divided by height in meters squared) who were accepted for bariatric surgery at participating surgical clinics in BEST were eligible for inclusion. No data on ethnicity were available because this information is prohibited to register in Swedish health care. Exclusion criteria were ongoing substance use disorder, uncontrolled psychiatric disease, inflammatory bowel disease, moderate to severe gastroesophageal reflux disease (unsatisfactory symptom relief with 20 mg/d of omeprazole), hiatal hernia larger than 4 cm, previous bariatric or other major upper gastrointestinal tract surgery, planned concomitant surgical procedure, or otherwise considered unsuitable for either of the 2 surgical techniques. Patient inclusion commenced October 6, 2015, and was completed March 31, 2022. The 30-day follow-up was completed for all participants in May 2022. Mortality at 90 days was assessed until August 15, 2022.

### Randomization

Patients were randomized to undergo either SG or RYGB using a 1:1 allocation with computerized randomization, except for the first 80 patients in Sweden (before a randomization module was in place) and all Norwegian patients (n = 102), for whom sealed envelopes were used. The randomization sequence used fixed block sizes of 10 and was stratified by center. Randomization was performed 24 hours before surgery at the earliest. Depending on the patients’ preferences, they were informed either before or after surgery. Patients were not blinded to the surgical procedure for safety reasons.

### Preoperative Evaluation and Preparation

Preoperative evaluation included laboratory blood analyses, quality-of-life questionnaires (RAND 36-Item Short Form Health Survey, Obesity-related Problem Scale, and the European Quality of Life 5-Dimension 5-Level Instrument), and routine preoperative workup. Routine preoperative endoscopy was initially only performed on clinical indication. However, after an increased appreciation of gastroesophageal reflux after SG,^19^ preoperative endoscopy was strongly recommended. All participants were prescribed a low-calorie diet for 2 to 4 weeks preoperatively.^40^

### Surgical Technique

A minimum annual center volume of 100 bariatric procedures was required, and for the individual surgeon, a minimum experience of 20 cases of each procedure was required. To minimize the learning curve of new surgeons, the local responsible investigator at each hospital was responsible for introducing new surgeons in BEST. Recruiting clinics submitted standard operating videos for review and approval.

The laparoscopic gastric sleeve included a vertical resection of the stomach along the curvature, using a 34- to 36-Ch bougie for calibration. The resection started 4 to 5 cm proximally from the pylorus and ended 1 cm from the angle of His. Buttressing or oversewing of the staple line was at the surgeon’s discretion.

The laparoscopic antecolic, antegastric RYGB included construction of a small gastric pouch (10-30 cm^3^). The gastrojejunostomy and jejunojejunostomy were created using a linear stapler and complementary hand-sutured closure of the remaining opening. Division of the greater omentum, unidirectional or bidirectional stapling of the jejunojejunostomy, and division of the mesentery between the gastrojejunostomy and the jejunojejunostomy were at the surgeons’ discretion and documented. The length of the Roux limb was recommended to be 100 to 150 cm, and the biliopancreatic limb was 50 to 75 cm. Mesenteric defects at the Petersen space and the jejunojejunostomy were closed with nonresorbable sutures or metal clips.^23^ Cruroplasty was recommended in patients with a hiatal hernia larger than 2 cm. Patients with a hiatal hernia larger than 4 cm, as estimated during the operation, were excluded, and surgery proceeded according to clinical judgment.

### Prophylaxis and Supplementation

Thrombosis prophylaxis and perioperative antibiotics were administered according to local routines. A proton pump inhibitor was prescribed to all participants for 30 days postoperatively (20 mg/d of omeprazole or equivalent) and thereafter on clinical indication. Vitamin and mineral supplementation was prescribed in accordance with the Nordic guidelines.^41^

### Outcome Measures

Perioperative complications were analyzed at 30 days, and mortality was assessed at 30 and 90 days after surgery with cross-reference with the Swedish Total Population Register^42^ (100% follow-up) and The National Population Register (Norway). Perioperative complications were analyzed both as any complication (adverse events) and as Clavien-Dindo grade IIIb or higher (serious adverse events).^43^ Serious adverse events were further analyzed according to type.

### Statistical Analysis

Data for patients who withdrew consent after randomization but before operation were not analyzed (n = 13). Continuous variables were analyzed with descriptive statistics (mean [SD] or median [IQR]). Numbers (percentages) were provided for categorical data. Differences between groups were evaluated with an unpaired *t* test for continuous variables and the Fisher exact test for categorical variables. For adverse events, a priori defined subgroup analyses were conducted by sex, age, diabetes status, baseline BMI, and smoking. Treatment interactions in subgroups were assessed with logistic regression models. All statistical tests were 2-sided, and *P* < .05 was considered statistically significant. Stata software, version 15.1 (StataCorp) was used for analyses.

## Results

**Figure 1.**
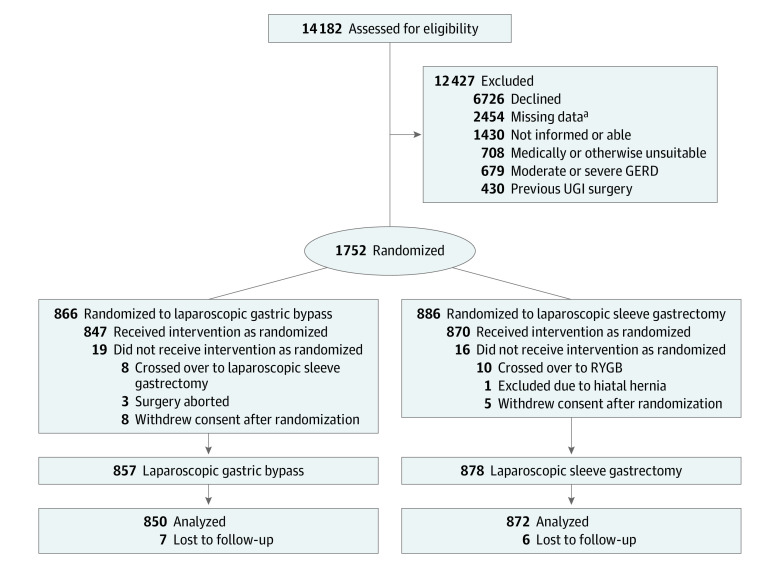
Flowchart for the Bypass Equipoise Sleeve Trial GERD indicates gastroesophageal reflux disease; RYGB, Roux-en-Y gastric bypass; UGI, upper gastrointestinal tract. ^a^Eligible but randomized without digital randomization; detailed exclusion data were not captured.

In BEST, a total of 1735 patients were operated on (1282 female [73.9%] and 453 [26.1%] male; mean [SD] age, 42.9 [11.1] years; mean [SD] BMI, 40.8 [3.7]), 878 with SG and 857 with RYGB. Twenty-three hospitals, 20 in Sweden and 3 in Norway, recruited and operated on patients in BEST, and between October 6, 2015, and March 31, 2022, 1752 of 14 182 eligible patients (12.4%) consented to participate in the trial (Figure 1). Of eligible but excluded patients in Sweden, 8552 (76.8%) were women, mean (SD) BMI was 41.2 (1.4), and mean (SD) age was 41.3 (11.7) years. The main reason for exclusion was patients’ preference of either method (eFigure 1 in Supplement 2).

Thirteen patients withdrew consent after randomization before operation, and 4 were excluded during surgery (1 due to a large hiatal hernia [>10 cm] and 3 for other reasons). Data for 1 excluded patient (large hiatal hernia) are included in the data on demographics but not in further analyses. The remaining 3 patients did not undergo bariatric surgery, and their data are not reported. Eight patients (0.5%) crossed over from RYGB to SG and 10 patients (0.6%) from SG to RYGB. One patient (0.1%) crossed over from RYGB to SG due to possibly compromised circulation, and 6 patients (0.3%) due to adhesions or short mesentery. Eight patients (0.5%) crossed over from SG to RYGB due to hiatal hernias (2-4 cm). In the remaining 3 patients (0.2%), the reasons for crossing over are unknown.

### Demographics and Surgical Data

Baseline characteristics are given in Table 1. There were no clinically relevant differences between groups at baseline.

**Table 1.** Demographic and Baseline Characteristics for Participants Randomized to Sleeve Gastrectomy or Roux-en-Y Gastric Bypass in the Bypass Equipoise Sleeve Trial^a^

Characteristic	Sleeve gastrectomy (n = 878)	Roux-en-Y gastric bypass (n = 858^b^)
Sex		
Female	660 (75.2)	622 (72.5)
Male	218 (24.8)	236 (27.5)
Weight, mean (SD), kg	117 (18)	118 (18)
Height, mean (SD), cm	169 (9)	170 (9)
BMI, mean (SD)	40.8 (3.7)	40.9 (3.8)
Waist circumference, mean (SD) cm^c^	123 (13)	125 (13)
Diabetes^d^	106 (12.1)	118 (13.8)
Dyslipidemia^d^	116 (13.2)	111 (12.9)
Hypertension^d^	251 (28.6)	259 (30.2)
Sleep apnea^d^	124 (14.1)	118 (13.8)
Dyspepsia^d^	36 (4.1)	42 (4.9)
Depression^d^	124 (14.1)	107 (12.5)
Prior DVT or PE	27 (3.1)	19 (2.2)
Smoking^e^	74 (8.5)	95 (11.1)

Abbreviations: BMI, body mass index (calculated as weight in kilograms divided by height in meters squared); DVT, deep vein thrombosis; PE, pulmonary embolism.

^a^ Data are presented as number (percentage) of patients unless otherwise indicated. With the exception of smoking and waist circumference, there are no missing values.

^b^ Includes patient that was excluded during surgery.

^c^ For waist circumference, data were missing for 27 patients in the sleeve gastrectomy group and 35 in the Roux-en-Y gastric bypass group.

^d^ The comorbidities diabetes, dyslipidemia, hypertension, dyspepsia, and depression were defined as using 1 or more pharmaceutical treatment or the use of continuous positive airway pressure machine for sleep apnea.

^e^ Smoking refers to current smokers with preoperative smoking cessation. Data were missing for 8 patients in the sleeve gastrectomy group and 4 patients in the Roux-en-Y gastric bypass group.

The SG resection started a mean (SD) of 4.5 (0.9) cm from the pylorus and ended 1 (0.5) cm from the angle of His. Any kind of staple line reinforcement was used in 419 of 878 patients (47.7%).

In RYGB, the gastric pouch was constructed using 45- and/or 60-mm cartridges to a mean (SD) total cartridge length of 149 (27) mm. The Roux limb and biliary limbs were a mean (SD) of 119 (18) cm and 58 (15) cm, respectively; closure of the Petersen space and the mesenteric defect at the jejunojejunostomy was performed in 844 of 846 patients (99.8%) and 843 of 844 patients (99.9%), respectively. The closure was performed with clips in 601 of 844 patients (71.2%) and with sutures in 243 of 844 patients (28.8%).

All RYGB and 877 of 878 SGs (99.9%) were performed laparoscopically. One patient receiving SG had adhesions and surgery was initially aborted. Open SG surgery was performed 4 months later. There were no other conversions from laparoscopy to open surgery (Table 2). The mean (SD) operating time was 47.3 (17.8) minutes in the SG group and 67.7 (25.3) minutes in the RYGB group (*P* < .001), with a median (IQR) operating time of 45 (36-56) minutes in the SG group and 65 (49-81) minutes in the RYGB group. Distribution of operating times is given in eFigure 2 in Supplement 2.

**Table 2.** Intraoperative and Perioperative Outcomes for Participants Randomized to Sleeve Gastrectomy or Roux-en-Y Gastric Bypass in the Bypass Equipoise Sleeve Trial^a^

Outcome		Sleeve gastrectomy (n = 878)	Roux-en-Y gastric bypass (n = 857)	*P* value
Operation time, mean (SD), min		47.3 (17.8)	67.7 (25.3)	<.001
Laparoscopic surgical access		877 (99.9)	857 (100)	>.99
Converted to open surgery		0	0	
Presence of a hiatal hernia^b^		39 (4.8)	45 (5.6)	.50
Axial length of hiatal hernia, mean (SD), cm^c^		2.0 (1.0)	2.3 (1.2)	.25
Intraoperative bleeding >100 mL		7 (0.8)	7 (0.8)	>.99
Intraoperative complications^d^		9 (1.0)	17 (2.0)	.12
Splenic injury		1 (0.1)	0	>.99
Bowel injury		0	9 (1.1)	.002
Other intraoperative complication		8 (0.9)	8 (0.9)	>.99
Thrombosis prophylaxis^e^	860 (99.9)		848 (99.8)	.62
Antibiotic prophylaxis^f^	858 (99.8)		847 (99.6)	.68
Postoperative hospital stay, mean (SD), d	1.3 (1.8)		1.3 (1.8)	.33
Readmitted to hospital within 30 d^g^	27 (3.1)		34 (4.0)	.33

^a^ Data are presented as number (percentage) of patients unless otherwise indicated. With the exception of values noted in footnotes, there are no missing values.

^b^ Data are missing for 63 patients in the sleeve gastrectomy group and 49 patients in the Roux-en-Y gastric bypass group.

^c^ Data are given for 38 patients in the sleeve gastrectomy group and 44 patients in the Roux-en-Y gastric bypass group.

^d^ Subsequent operations within 30 days are not included (Figure 2).

^e^ Data are missing for 17 patients in the sleeve gastrectomy group and 7 patients in the Roux-en-Y gastric bypass group.

^f^ Data are missing for 18 patients in the sleeve gastrectomy group and 7 patients in the Roux-en-Y gastric bypass group.

^g^ Data are missing for 5 patients in the sleeve gastrectomy group and 6 patients in the Roux-en-Y gastric bypass group.

The median (IQR) postoperative hospital stay after both SG and RYGB was 1 (1-1) day. All patients received antibiotic prophylaxis. Thrombosis prophylaxis with low-molecular-weight heparin was given; in 1550 of 1711 patients (90.6%), the higher dose was administered (dalteparin, 5000 IE or equivalent).

### Adverse Events

**Figure 2.**
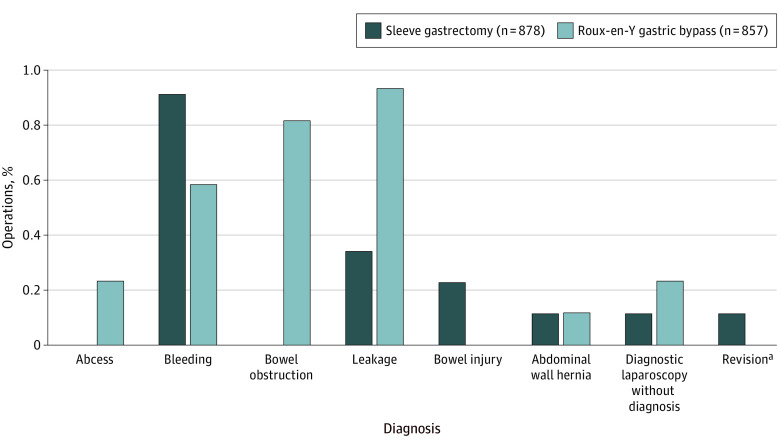
Diagnoses at Reoperation for Complications Until 30 Days Postoperatively After Sleeve Gastrectomy and Roux-en-Y Gastric Bypass Intraoperative complications are not shown (Table 2). The total numbers of patients undergoing subsequent operations were 14 for sleeve gastrectomy and 23 for Roux-en-Y gastric bypass (some patients had >1 diagnosis at reoperation). One subcutaneous abscess was incised with the patient under local anesthesia. The 2 patients who underwent gastroscopy in general anesthesia are not shown. ^a^Revision surgery indicates sleeve gastrectomy revised to Roux-en-Y gastric bypass.

**Figure 3.**
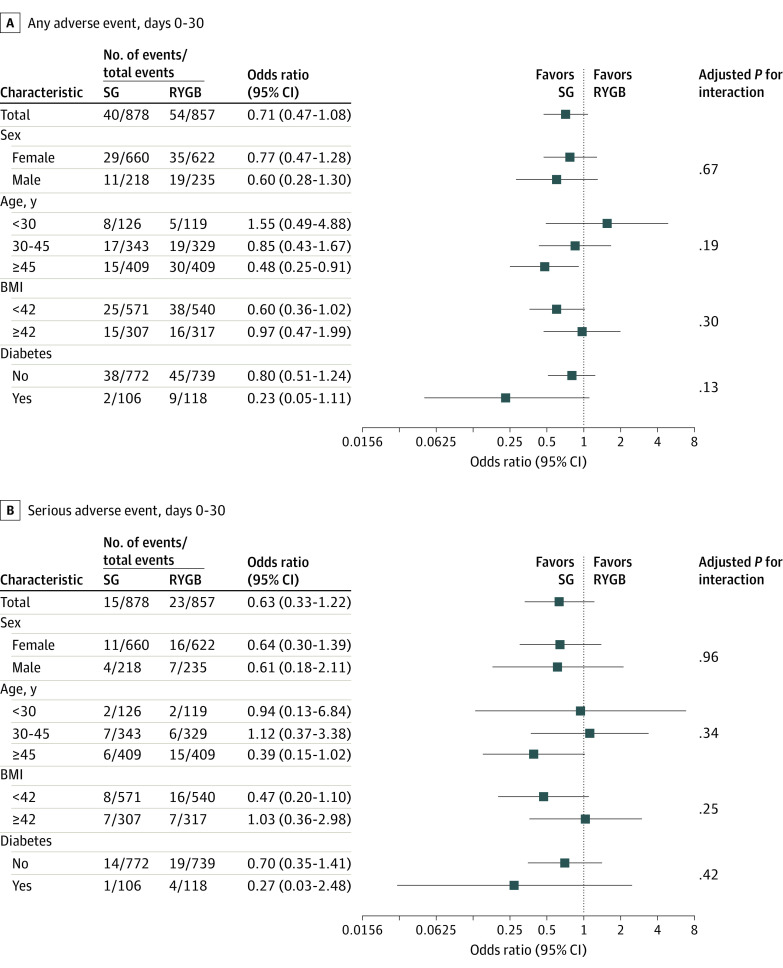
Risk (Adjusted Odds Ratio After Multivariate Logistic Regression) of Any Adverse Event and Serious Adverse Events There were no statistically significant differences between sleeve gastrectomy (SG) and Roux-en-Y gastric bypass (RYGB) in these analyses. BMI indicates body mass index (calculated as weight in kilograms divided by height in meters squared).

There was no 30- or 90-day mortality. Any adverse event occurred in 40 of 878 patients (4.6%) in the SG group and 54 of 857 patients (6.3%) in the RYGB group (*P* = .11). The odds ratio (OR) for any adverse event comparing SG with RYGB was 0.71 (95% CI, 0.47 to 1.08; absolute risk difference, −1.7 percentage points; 95% CI −3.9 to 0.4 percentage points). A serious adverse event occurred in 15 of 878 patients (1.7%) in the SG group and 23 of 857 (2.7%) in the RYGB group (OR, 0.63; 95% CI, 0.33-1.22; absolute risk difference, −1.0 percentage points; 95% CI, −2.4 to 0.4 percentage points; *P* = .19). The only significant difference of serious adverse events between the groups was for small bowel obstruction, occurring in 0 of 872 patients (0%) in the SG group and 6 of 850 (0.7%) in the RYGB group (*P* = .01). There were 41 complications registered at subsequent operation in 37 patients (Figure 2). There were no adverse events with a Clavien-Dindo grade higher than IIIb. There was no interaction in the risk of complications at 30 days between SG and RYGB in the predefined subgroups (sex, age, BMI, diabetes, and smoking) (Figure 3).

Sixty-one patients (27 of 873 [3.1%] in the SG group and 34 of 851 [4.0%] in the RYGB group; *P* = .33) were readmitted during postoperative days 0 to 30. The median (IQR) length of hospital stay during the second admission was 3 (1-4) days and 2 (1-4.25) days for SG and RYGB, respectively. The main reason for readmission was pain or dehydration in both groups (eFigure 3 in Supplement 2). Variation in adverse events, operating time, and in-hospital stay per hospital are presented in the eTable in Supplement 2.

## Discussion

In this large randomized clinical trial comparing SG and RYGB, we found low rates of perioperative complications without statistical significance between groups. Gero et al^44^ suggested that an early (<90 days) severe complication rate (Clavien-Dindo grade IIIa or higher) of less than 5.5% is a reasonable benchmark for low-risk bariatric surgery with SG and RYGB. Although this article reports 30-day data, it appears likely that the complication rates of Clavien-Dindo grade IIIb or higher in BEST of 1.7% for SG and 2.7% for RYGB are well in line with international bariatric surgical quality standards. This finding is noteworthy considering that BEST was performed in a pragmatic multicenter setting, not restricted to low-risk patients or high-volume centers. However, similar complication rates have been reported previously.^45,46^

The absolute proportion of serious adverse events within 30 days in this trial was higher in the RYGB compared with the SG group, although this finding was not statistically significant. This difference aligns with previous randomized studies, such as Sleeve vs Bypass trial (SLEEVEPASS)^47^ and Swiss Multicenter Bypass or Sleeve Study (SM-BOSS),^48^ in which serious complication rates after SG and RYGB were 5.8% vs 9.4% and 1% vs 4.5%, respectively, and is further supported by reports from large databases.^12,13^ A small difference in perioperative risk favoring SG could be assumed to be of limited clinical relevance, especially in the view of other factors, such as long-term weight control and effect on comorbidity.

Although there is a small difference in favor of SG regarding complications between the groups in BEST, this difference is smaller than reported from large database studies from the US.^12,13^ A larger risk difference for complications between groups in nonrandomized studies may in part be due to selection bias, in which healthier patients are more likely to undergo SG. However, patients in the Metabolic and Bariatric Surgery Accreditation and Quality Improvement Program (MBSAQIP) study^13^ had a higher mean BMI and higher prevalence of comorbidities than in BEST, which likely is associated with more challenging surgical conditions and may therefore be more differentiated between simple and complex bariatric procedures.

The comparatively low complication rate in the RYGB group in BEST may be associated with the fact that laparoscopic RYGB has been the gold standard procedure for decades in Scandinavia. There is, furthermore, a strong tradition of open reporting of complications and various aspects of surgical technique in national conferences. Additionally, SOReg has been pivotal in facilitating standardization of surgical technique, which has likely further contributed to a reduced risk of complications over time.^46,49^ Data from BEST indicate that a surgical community with wide experience performing RYGB can change to performing SG with low complication rates. However, whether the opposite transition can be safely adopted remains to be demonstrated.

Small bowel obstruction was more common in BEST than in previous reports on perioperative complications after RYGB.^47,48,50^ The higher risk of small bowel obstruction may be related to the Lönroth surgical technique for RYGB,^51^ in which closure of mesenteric defects have been demonstrated to be associated with an increased incidence of small bowel obstruction during the first year after surgery, although the long-term risk of small bowel obstruction was markedly reduced.^23^

The operating time was longer for RYGB compared with SG, which is in accordance with data from previous randomized clinical trials^47,48^ and likely reflects the higher degree of surgical complexity in RYGB. The length of postoperative hospital stay was only 1 night after surgery, without a difference between SG and RYGB, which is consistent with previously published data.^12,47^

### Strengths and Limitations

A quality registry-based randomized trial design includes several advantages, such as an existing infrastructure for data capture and an excellent 30-day follow-up rate. Assessment of external validity by comparison with nonrandomized patients in the registry is also possible. There is, however, a limitation as to how much data are realistic to capture in routine clinical practice and possibly a higher risk of attrition, although this can be mitigated through capture of data in other relevant national health care registries. Other strengths of BEST include the multicenter, pragmatic design with a large sample size. A prespecified per-protocol analysis of perioperative data represents a clinically relevant perspective for surgical safety comparing SG and RYGB.

A potential limitation of the generalizability in BEST is that only 12% of patients with basic eligibility (BMI and age) consented to inclusion, although this is not uncommon in randomized surgical trials.^32^ Demographic data in excluded patients do not suggest a systematic selection bias, and most exclusions were at the patient’s request. A possible limitation is in the upper BMI limit of 50; although this covers most bariatric patients in Scandinavia, we recognize that it may limit generalizability to other populations. Another potential disadvantage is that a standardized antecolic, antegastric RYGB construction was used, which may limit the generalizability to surgical communities using other techniques for RYGB, as well as the fact that no data regarding ethnicity were given (prohibited to register in Swedish health care). Although a subanalysis was performed regarding outcome by sex, the large proportion of women in BEST could still be viewed as a limitation. A potential limitation is that 4 of 23 hospitals (17%) recruited more than 60% of all participants, although this variability in center volumes is representative of bariatric practice in Scandinavia. Some hospitals, including those in Norway, joined BEST late during the inclusion period and therefore contributed fewer patients. The sample size in BEST is based on a power calculation for the primary end points (weight loss and substantial adverse events at 5 years) constituting a limitation to this study, as can be noted by the wide 95% CIs of adverse events.

## Conclusions

We conclude that among adult patients in Sweden and Norway with a BMI of 35 to 50 who underwent SG and RYGB in a large randomized clinical trial, the perioperative morbidity was low and not significantly different between the groups. We therefore suggest that the perioperative risk should be of limited focus in the choice between SG or RYGB.

## Notes

Supplement 1.
Trial Protocol, Statistical Analysis Plan, and Perioperative Statistical Analysis Plan

Supplement 2. **eFigure 1.** Reasons for Exclusion Among Eligible Patients Being Asked About Inclusion in the Randomization Module in SOReg **eFigure 2.** Distribution of Operating Time According to Procedure. Sleeve Gastrectomy (SG), Roux-en-Y Gastric Bypass (RYGB) **eFigure 3.** Reasons for Readmission for the 61 Patients Readmitted Within 30 Days **eTable.** Complications, Operation Time and In-Hospital Stay, Analyzed by Participating Hospitals, in Patients Randomized to Sleeve Gastrectomy and Roux-en-Y Gastric Bypass in the Bypass Equipoise Sleeve Trial (BEST)

Supplement 3.
Nonauthor Collaborators

Supplement 4.
Data Sharing Statement
