# Error in Author Affiliations

**DOI:** 10.1001/jamanetworkopen.2024.8600

**Published:** 2024-03-28

In the Original Investigation titled “Comparison of Sleeve Gastrectomy vs Roux-en-Y Gastric Bypass: A Randomized Clinical Trial,”^1^ published January 30, 2024, an affiliation was missing for author Torsten Olbers; Dr Olbers is affiliated with the Department of Biomedical and Clinical Sciences, Linköping University, Linköping, Sweden, in addition to the Wallenberg Centre for Molecular Medicine, Department of Biomedical and Clinical Sciences at Linköping University and the Department of Surgery, Vrinnevi Hospital, Norrköping, Sweden. This article has been corrected.^1^
