# Supplementary material for: Comparison of Sleeve Gastrectomy vs Roux-en-Y Gastric Bypass: A Randomized Clinical Trial
Source: JAMA Netw Open. 2024 Jan 30;7(1):e2353141. doi: 10.1001/jamanetworkopen.2023.53141 (PMC10828911; doi:10.1001/jamanetworkopen.2023.53141)
Supplement: Supplement 1. — Trial Protocol, Statistical Analysis Plan, and Perioperative Statistical Analysis Plan [file jamanetwopen-e2353141-s001.pdf]

STUDY PROTOCOL

BEST- Bypass Equipoise Sleeve Trial

Bypass or Sleeve; Randomized controlled multicenter trial comparing Roux-en-Y gastric bypass and sleeve gastrectomy

TABLE OF CONTENTS

Background 3
The study 5
Participating Clinics 7
Patient recruitment 8
Operating methods 9
Gastric bypass 9
Sleeve gastrectomy 9
Identification of hiatus hernia or other aggravating circumstance perioperatively 10
Gastroesophageal reflux disease and gastroscopy 10
Other treatment 10
Data management and Statistical analysis plan 11
Management of the study 11
Schedule 12

Background

Laparoscopic Roux-en-Y gastric bypass (RYGB) has in recent years been the standard operation in Swedish bariatric surgery. The operation is well studied and we have a good idea of the expected result regarding weight, BMI, comorbidities, and it can be performed with a low complication rate. However, this does not mean that the surgical method is without challenges, and above all there is a small but important group of patients with prolonged morbidity after the procedure. Due to the bypassing of the duodenum and proximal jejunum, there is also a concern for long-term risk of various types of deficiency conditions.

Laparoscopic sleeve gastrectomy (SG) is a relatively recently introduced surgical method that in recent years has gained rapid and wide spread internationally and is currently the most commonly used obesity surgical technique in the United States. However, there are not results from long-term studies to the same extent as for RYGB. However, the reported data indicate good results regarding weight development and resolution of comorbidity up to a few years after the operation, although to a somewhat lesser extent compared to after RYGB. There are currently no long-term data presented for SG.

RYGB has been associated with a non-negligible risk of internal herniation with small bowel obstruction, which appears to affect at least 10% of patients operated on without closure of mesenteric defects, and despite closure a risk remains. This risk of internal herniation does not exist with SG. Another advantage of SG is that it avoids the problems with the Jejunojejunostomy (JJ), which seems to be part of the rare but difficult to treat morbidity after RYGB. There is also probably a slightly lower risk of vitamin and mineral deficiency because the duodenum and proximal jejunum are not bypassed. However, there are data that suggest that problems with gastro-oesophageal reflux may occur or worsen after SG and that continuous acid-suppressing treatment may be necessary. There are also indications that revisional surgery rates may be higher after SG, e.g. due to unsatisfactory weight development over time.

Today, therefore, there is no scientifically based unequivocal answer to which surgical method is best for the individual patient. The choice of surgical method is often based instead on local/regional guidelines and traditions, and sometimes on the patient's wishes or the surgeon's preference. There is therefore a great need for data that can provide information on how the outcome differs between operations and, in particular, if there are differences regarding outcomes in different subgroups of patients. This information is of great value to the healthcare provider as well as surgeons and patients. Internationally, there are a number of ongoing studies that compare RYGB and SG, however with a limited sample size (n=100–200) and where, as a rule, weight loss was chosen as the primary outcome variable. Existing data therefore do not allow comparisons between the surgical methods within different subgroups of patients (eg male/female, younger/middle-aged, +/- different types of comorbidity).

Scandinavian obesity surgery has a unique position and opportunity to answer the question: which of RYGB or SG should be recommended. The patient base in Sweden is relatively large with 5–6000 operations annually and with a national quality register - SOReg - with a 97.9% coverage rate. In Norway, approximately 3,000 operations are

carried out annually. With the help of social security numbers and data from national health registers at the National Board of Health and Welfare and other authorities, in Sweden we can plan for a complete long-term follow-up regarding factors such as health care consumption (Patient Register and Medical Register), working ability (Försäkringskassan), diabetes development (Medical Register and National Diabetes Register (NDR)) and cancer incidence (Cancerregistret). In Norway, we can use the cause of death register, cancer register, prescription register, SSB and NPR data to obtain a complete long-term follow-up.

Bariatric surgery in Sweden and Norway is characterized by great consensus and openness between operating units. In the internationally recognized obesity surgery study SOS (Swedish Obese Subjects), basically all patients who underwent obesity surgery in Sweden over a 15-year period (n=2010) were included and followed, which has led to decisive publications that strongly position Scandinavian bariatric surgery.

Surgically, there is also agreement regarding technical aspects of how bariatric surgery should be carried out, where in principle all surgeons perform a laparoscopic gastric bypass with antecolic antegastric technique according to the Sahlgrenska model. There is also great consensus regarding the technology for the sleeve. There is great openness in sharing results and clinical experiences, which has been facilitated to a large extent with the help of good registration in SOReg and joint complication conferences. In Norway, sleeve gastrectomy has been introduced at a rapid pace and most centers today offer both methods to their patients. The methods are largely standardized in the same way as in Sweden.

The study

We want to compare the outcome of RYGB and SG in a large study that includes several outcome variables in addition to weight control, and with a sufficiently large number of patients to allow comparisons within different subgroups (specification see below). This is done via a national registry-based randomized controlled multicenter trial where patients are randomized to RYGB or SG. In this way, we obtain unique data that will contribute to being able to define what role SG and GBP should have in future Scandinavian and international bariatric surgery.

Follow-up of the patients will take place in the regular structure for follow-up in SOReg after 6 weeks, 1 year, 2 years, and after 5 years. In addition, via SORreg and registry studies, we want to follow the patients for the rest of their lives. Through good baseline characteristics of the patients and planned follow-up with coordination of quality registers and other national registers, we expect to be able to achieve close to 100% follow-up of the patients regarding various outcome measures.

It should be noted that the Ethics Review approved lifelong follow-up in registers, but that the first patient information states that the code key is destroyed 10 years after the end of the study. Thus, new consent may be needed to look at 15-year data and beyond, this should be investigated in good time before such a follow-up becomes relevant.

Primary outcome measure

To evaluate whether SG has advantages compared to previous standard GBP, we want to investigate whether the surgical methods are equivalent (non-inferiority) regarding weight loss and weight stability five years after the operation, and whether SG is associated with fewer long-term complications (superiority). The primary outcome measure is thus based on a balanced assessment of long-term weight control and the frequency of serious complications.

Serious complications (Substantial Adverse Events) are defined as:

- Death caused by complication of bariatric surgery.
- Intervention in general anesthesia (Clavien-Dindo 3b and higher) for complication to bariatric surgery. Among these, gallstone interventions, hernias that were present before the primary operation and plastic surgery due to excess skin are excluded. Subgroup analysis of negative laparoscopies is planned.
- Intensive care as a result of a complication to obesity surgery
  Revision surgery (change of surgical method, reversal or significant change of original method) Revision surgery is performed according to clinical practice. The indication for revision surgery, e.g. unsatisfactory weight development or possible occurrence of GERD must be documented.
- Hospital care for an operation-related complication (apart from observation less than 1 day)
- Need for enteral (via tube to the GI tract) or parenteral total/supplemental nutrition
- Chronic abdominal pain (> 6 months) that requires regular (several times per week or daily) medication of opiates (ATC code N02A, including Tramadol and

Codeine) and/or abdominal pain that significantly affects daily life (prevents normal work, leisure activities, etc.)
- Frequent and severe meal-related problems that affect the normal way of life for at least 3 months (eg hypoglycaemia, pain, vomiting, nausea).
- Hypoglycemia associated with loss of consciousness/convulsions or requiring drug treatment
- Gastro-oesophageal reflux problems that cannot be controlled with medication (including volume reflux). Barrett's esophagus (in cases where you had a preoperative normal endoscopy within 2 years before the op).
- Clinically relevant, symptomatic deficiencies of vitamins and/or minerals.
- Severe anemia; Hb <100 g/L in women and <110 g/L in men.
- Other serious complication that is not predefined but after assessment by the reference group is judged to be surgery-related

Information regarding complications is collected during visits and registered in SOReg where a number of complications are listed but where there is also a field for "other". The seriousness of the complications is assessed continuously and finally after 5 years (primary endpoint). In case of doubt, the severity of the complication is determined by a reference group, which consists of 3 experienced bariatric surgeons (Ingmar Näslund, Johan Ottosson, Erik Stenberg).

In the calculations for long-term complications (5 years), i.e. patients who have undergone GBP and who incur some kind of serious complication (need for surgery for complication, revision surgery, serious nutritional problems, or chronic abdominal pain), it has been shown during the course of the study that the number of substantial AE (adverse events) is significantly higher than we initially expected. This has made it possible, after renewed power calculations based on new data, to reduce the number of included patients, ie. see Statistical analysis plan v 5.

Secondary outcome measures
Secondary outcome measures include health economics, inpatient care, outpatient care, drug consumption, alcohol abuse, fractures, diabetes, cancer, mortality and quality of life. Secondary outcome measures are described in greater detail in the statistical analysis plan.

As a comparative measure of quality of life, patients will complete the EQ5D-5L, SF-36 and OP quality of life questionnaires at baseline, as well as at 1 year, 2 years and 5 years. In Norway, the validated quality of life questionnaires WRSM, IQOQOL, HADS, GSRS and GERDQ are also filled in. To look at quality of life related to alcohol, we also want to follow/analyze AUDIT and B-Peth, where those parameters are available.

The physical examinations (abdominal circumference, etc.) that are done and blood samples that are taken in the study at 1- and 2-year controls should take place in the time interval -1 month to +3 months around the target date. For the 5-year check-up, the time interval ± 6 months around the target date is accepted.

For data verification and completion, information regarding the bariatric surgery and background information can be retrieved from medical records.

The mandatory analyzes are vitamin D, P-Glucose and HbA1c. In Norway, a standard set of blood samples is taken in addition to the mandatory analyzes in SOReg.

We are planning subgroup analyzes regarding sex, age (18–29; 30–45; >45 years), BMI (<42 and >42kg/m ^{2} respectively, which is the median BMI in SOReg) and +/- type 2 diabetes.

Subgroup analysis regarding gallstone intervention is planned.

In addition to the clinical follow-up, we will, with the help of coordination between SOReg and other national registers(Medicines register (2005-), Patient register (inpatient care 1987-, Outpatient care 2001-); The Cancer Registry, Intensive Care Registry, National Diabetes Registry, Försäkringskassan's MiDAS register, the Swedish Tax Agency and other relevant national registers), get a good picture of the effect, safety and cost-effectiveness of each surgical method over a long follow-up period. In Norway, the corresponding national registers will be used (the Cancer Register, the Cause of Death Register, the Prescription Register, NPR data and data from Statistisk Sentralbyrå).

Which correlations are to be made and for which variables are specified in the statistical analysis plan for each sub-study, as well as in applications for data extraction from each register, in accordance with the respective laws and regulations in Sweden and Norway.

Participating Clinics

Participating clinics in Sweden and Norway must register in SOReg.

The clinic must have a volume of at least 100 bariatric surgeries/year. To be certified as a BEST clinic, you must have performed at least 20 operations each of GBP or SG as well as having sent in a film of one type of procedure of each technique. Each clinic has a local PI who sends type-films to the trail management to verify agreement in method between participating operators and clinics. The type films are after February 2017 are verified blindly with respect to the clinic. The videos will be deleted when the review is complete.

Patients who are not included in the study are treated according to the clinic's usual treatment strategy.

Through regular joint web-based conferences, we will be able to verify consistency in assessments and surgical technique.

In addition to the usual mandatory parameters in SOReg, participating centers undertake to also register the otherwise voluntary parameters (e.g. smoking, level of

education, weight at surgery, blood pressure, quality of life) and to take the blood samples recommended in SOReg.
For the health economic analysis, EQ5D-5L is used. This is filled in by the patients at baseline, as well as after 1 year, 2 years and 5 years.

A financial compensation is paid to participating clinics (currently only in Sweden) of SEK 800 per randomized patient and SEK 400 for the patient's 2- year follow-up. Our ambition is also to be able to finance five regional coordinators with the help of granted research funds to improve the follow-up rate at the 2- and 5-year examinations, or contribute in a similar way to an improved follow-up rate. The costs are initially assessed to be equivalent between the two surgical methods.

Patient recruitment

The prospective study participant/patient is informed about the study in connection with an visit for bariatric surgery, following the respective clinic's usual referral procedure. Written and verbal information regarding the background of the study and the two surgical methods is given. If the patient is willing to participate, written informed consent is obtained in connection with the clinic visit or enrollment depending on local routines.

In connection with the start of the study at participating centers, if necessary, a member of the steering group will participate and answer questions in connection with patient information and provide both written study information and slideshows. A website was created for additional patient information from which you can also get in touch with the study management, beststudien.se. This website will end at the turn of the year 22/23, but some information will in the future be available on SOReg's website.

Inclusion criteria:
- >18 years of age at inclusion.
- BMI 35–50 kg/m^2, before preoperative weight loss.
- Otherwise approved for obesity surgery according to the clinic's routines.
- Must be able to understand the information, and to make an informed decision regarding, participation in the study.

Exclusion criteria:
- The patient is deemed not suitable for surgery with either method according to the surgeon's assessment or the clinic's routine
- Ongoing substance abuse. (Requires documented drug abstinence according to the clinic's routines, but minimum 1 year)
- Unstable psychiatric illness or other known contraindication to bariatric surgery .
- Previously undergone bariatric surgery or antireflux surgery.
- Moderate-severe reflux disease. (Not symptom-free on PPI in a dose equivalent to 20mg Omeprazole/day, and/or volume reflux, or presence of glandular metaplasia (Barrett's oesophagus)) or known hiatal hernia >4 cm
- Planned other simultaneous significant surgery, e.g. cholecystectomy.
- Inflammatory bowel disease

Randomization

Initially envelope-randomization was used in the trial. Since spring 2016 randomization is conducted via a module in SOReg, which creates a computer-generated randomization. If there is a problem with access to SOReg at the time of randomization, you should call the central study team BEST who can provide envelope-randomization, alternatively you toss a coin for randomization. It is important that the central study team BEST is informed about the randomization outcome so that it can be entered into the SOReg/system.

Randomization takes place on the morning of the day of surgery to allow for preparation in the operating room, and the patient is informed of the surgical method postoperatively. For those patients who wish to be informed about the randomization result before surgery, this can be shared up to 24 hours before anaesthesia. In order to avoid the patient choosing to withdraw from the study due to disappointment with the randomization outcome, it is important that the person giving the information makes sure that the patient is still willing to be randomized before the actual randomization is carried out. The randomization is done in blocks of 10 patients.

In Norway, randomization takes place in a separate randomization program developed for operating Norwegian clinics.

Operating methods

Data relating to the surgery are documented in SOReg, in addition to the operation notes in the patient's medical record.

Gastric bypass

Laparoscopic antecolic antegastric gastric bypass according to the so-called Sahlgrenska model.

Linear stapled and hand-sewn gastroejunostomy. Some clinical experience suggests that division of the small bowel mesentery between the anastomoses to the first arcade may be of value in reducing kink-formation. If mesentery division is performed must be documented. Which direction stapeling of the JJ occurs and whether it is uni- or bidirectionally stapled is documented. Mesenteric defects are closed with clips. (1 or 2 rows) or with a non-absorbable continuous suture, not a “tobaco-pouch” suture in conjunction to the JJ. Mesenteric defects should be closed deep in the mesentery with care to avoid kinks at the JJ. The biliopancreatic limb should be 50-100cm and Roux bone 70-150cm.

Sleeve gastrectomy

The sleeve must be formed without pressure, using a bougie (35-36 Ch) as guide. The surgeon can freely choose types cartridges for stapeling, but which ones have been used must be documented. The resection is started at a distance of 4-6 cm from the pylorus and ends maximum 1 cm from the angle of His. Special care is taken not to create a constriction of the tube at the angulus. The surgeon chooses whether any reinforcement

of the staple line should take place (suture alt buttress). Whether this happens or not must be documented.

Identification of hiatus hernia or other aggravating circumstance perioperatively

If the hiatus hernia is identified intraoperatively, this must be documented. The minimum for dissection is to expose the left crus. Hernias between 2-4cm may be repaired with cruroplasty. In the case of a hernia >4cm, the patient is excluded from the study and the surgeon is free to continue in the way that is considered clinically best.

Gastroesophageal reflux disease and gastroscopy

The study management recommends that gastroscopy be performed on patients planned to be included in BEST to rule out the presence of a larger hiatal hernia, severe esophagitis or suspected Barrett's esophagus, which in most cases constitute a contraindication for SG

The gastroscopy can be done in a separate session preoperatively or under anesthesia at the start of the operation, **but note in the latter option that the patient must not be randomized until after the gastroscopy and therefore cannot be informed about the surgical method before anaesthesia**.

During the course of the trial, there have been indications of an increased risk of esophagitis and Barrett's esophagus, especially after gastric sleeve. International and upcoming national guidelines recommend gastroscopy after 5 years for all sleeve patients. Based on this, the BEST steering group decided in spring 2021 that gastroscopy should be performed 5 years after surgery on all patients included in BEST. Study plan, ethics application and patient information have been updated/supplemented. The ethics application was approved in autumn 2021. Study participants are thus asked for separate consent for gastroscopy. If gastroscopy was carried out on clinical indication within the time frame +/- 1 year from the five-year check-up, information about this can be obtained and replace study-specific gastroscopy.

Other treatment

Thromboprophylaxis is given according to the clinic's routine.

All included patients are prescribed PPI postoperatively (Ome-/Pantoprazole 20mg/day for 1 month). After that, if necessary, based on clinical indication.

Antibiotic prophylaxis must be given. Choice of substance and method of administration are according to the clinic's routines.

All patients are prescribed supplementation with multivitamins, calcium/vitamin D and vitamin B_{12} and, if necessary, Fe, in accordance with the Nordic guidelines [1].

[1] http://www.ucr.uu.se/soreg/dokument/nordiska-riktlinjer-foer-monitorering-och-supplementering-med-vitaminer-mineraler-samt-upfoeljning-efter-obesitaskirurgi

Data management and Statistical analysis plan

SOReg constitutes the "Case Report form (CRF)" and during the data collection phase the trial follows SOReg's rules and procedures. Papers relating only to the study (Informed Consent, EQ5D, etc) are archived with the study director/principal.

Agreements are drawn up on the sharing of data, based on the needs of participating researchers/principals to be able to carry out the study according to plan.

Register withdrawals from national registers are applied for according to respective registry's routines and based on the approved ethics application.

For further information please refer to the Statistic Analysis plan (version 5), the published method manuscript, and a separate data handling and analysis plans for each publication.

Management of the study

A management office for the study is established at Sahlgrenska University Hospital in Gothenburg (VGR), Sweden.

Participating clinics are free to conduct substudies on their own patient material and therefore we will randomize in blocks so that an equal number of procedures with each technique are performed at each clinic. However, such studies need to be communicated and discussed with the study management, to avoid duplicate projects and facilitate coordination if several centers are planning similar projects. Additional projects/studies must comply with current legislation with supplementary patient information and ethics application if necessary.

Publication plan

Planned main publications are estimated to be:

- Peri-operative results: 30-day results, mainly complications
- Two-year results: weight and adverse events
- Five-year results: weight and adverse events
- Five years: cost-effectiveness

We plan that one person from each center that contributed to the inclusion of at least 100 people will be invited to be a co-author. The aim is to publish under "On behalf of the BEST study group", and all authors are listed and credited in the work. However, for the final decision of the order of authors, the journal's guidelines must be taken into account.

Trial meetings

Once a year, participants at all clinics are invited to an investigator's meeting where information about the progress and results of the trail are presented and questions can be discussed.

Schedule

Nov 2014-August 2015	Preparations, study protocol, steering group, ethics application, etc.
Sept-Oct 2015	Inclusion start
Autumn 2018	Inclusion start in Norway
31 March 2022	Inclusion complete

Statistical analysis plan for:

BEST- Bypass Equipoise Sleeve Trial

Randomized controlled Scandinavian multicentre study comparing gastric bypass and sleeve gastrectomy

version 5; Date: September 12th 2022

Acronym: BEST

Clinical Trial Registration Number: NCT02767505

Principal Investigator: Torsten Olbers

Senior statistician: Markku Peltonen

Independent statistician: Max Petzold

1st version of analysis plan prepared by: Markku Peltonen, PhD

Project start date: October 1st, 2015

Expected completion date: All 5-year data collected at latest Oct 31st 2027.

Contents

1. Introduction 3
2. Study objectives 3
3. Study Design 3
    3.1 Overview 3
    3.2 Randomization 4
    3.3 Eligibility criteria 4
    3.4 Data collection and follow-up 4
4. Study outcomes 5
5. Statistical methods 8
    5.1 Sample size and power 8
    5.2 Principles of analysis 8
    5.3 Statistical analyses 9
    5.4 Study follow-up and missing data 10
    5.5 Timing of analyses 10
Appendix: 11

1. Introduction

Gastric bypass (GBP) has been the standard surgical intervention to treat severe obesity in Sweden. Sleeve gastrectomy (SG) is a more recently introduced surgical technique, which has been used increasingly both in Sweden and internationally. Data from some smaller RCTs report no major differences between SG and GBP in terms of weight loss or resolution of comorbidities at short- and median-long follow up (Peterli, Salminen). However, the long-term effects of SG on body weight and health, as well as on complications and adverse events are less well described.

The current investigator-initiated study is a randomized, parallel group, Scandinavian multicentre study comparing long-term health effects and complications of GBP and SG. The study aims to evaluate a potential safety advantage of SG over GBP, and makes an efficacy comparison between the two treatments.

2. Study objectives

The objective of the study is to evaluate the safety and effectiveness of SG compared to GBP in obese patients eligible for bariatric surgery.

The study has two primary objectives, evaluating superiority and non-inferiority. The study hypothesis for superiority is that patients treated with SG will experience fewer adverse events during the 5-year follow-up as compared to patients treated with GBP.

For non-inferiority, the study hypothesis is that the weight loss in the SG group, as compared to GBP, is within a non-inferiority margin of 5% at 5 years after surgery.

Secondary objectives of the study include health economics, inpatient hospital and outpatient care, drug consumption, alcohol abuse, fractures and bone quality, diabetes, cancer, mortality, and quality of life.

Predefined subgroup analyses will be done by sex, age, diabetes status and body mass index (BMI).

3. Study Design

3.1 Overview

This is a randomized, Scandinavian multi-centre study comparing the two most abundant bariatric surgery techniques worldwide; laparoscopic gastric bypass (GBP) and laparoscopic sleeve gastrectomy (SG). Study design is parallel group study with 1:1 allocation between GBP and SG.

To be invited to participate in the study as a collaborating centre, the operating bariatric clinic has to perform at least 100 bariatric surgeries annually, report to the Scandinavian Obesity Surgery Registry (SOReg) and be able to meet all criteria set in the project plan. The study, to be conducted at approximately 20 bariatric surgery clinics in Sweden and Norway, will include 2100 obese patients approved for bariatric surgery (1050 patients to be operated with SG and 1050 with GBP).

The follow-up time for the primary objectives is 5-years.

3.2 Randomization

The randomisation sequence was generated using Stata version 12.1 with a fixed block size of 10. Patients are randomized in the ratio of 1:1, stratified by the participating bariatric surgery clinics (centers).

The results of randomization will be provided centrally using computerized system and cannot be affected by individual operating clinics.

The surgeon performing the operation will be aware of group allocation at maximum one day before the actual operation. If the patient choses, he/she will be informed the day before, or on the morning of the day of surgery, whereas those who do not wish to be informed preoperatively will be informed postoperatively about the surgical method used. For safety reasons all patients will be informed about the surgical method used. Informed consent is given before randomization by all participants.

3.3 Eligibility criteria

The target group is adult (>18 years) patients with severe obesity (BMI 35-50 kg/m^2) eligible and approved for bariatric surgery in Sweden or Norway. To be included in the study, patients need to fulfil the inclusion criteria, not having any exclusion criteria, and be eligible for operation using both surgical techniques. Detailed inclusion and exclusion criteria are provided in the study protocol.

3.4 Data collection and follow-up

Baseline data regarding demographics and health parameters will be collected by the operating clinics using standardized questionnaires and measurements. The data collection is facilitated using a central computerized system within the Scandinavian Obesity surgery Registry - SOReg.

Clinical health examination of the patients including data on body weight will be conducted at 6 weeks, 1 year, 2 years, and 5 years after surgery.

Follow-up regarding substantial adverse events will be conducted using clinical information as well as by using Swedish and Norwegian national health registries (see below). These registries have, in general, a close to complete coverage. Individual patients can be monitored using the unique personal identification number assigned to every person residing in Sweden and Norway.

In order to analyze data for primary and secondary objectives, data retrieved from SOReg will be linked by personal identification number to the following registers: Swedish Patient Registry; Prescribed Drug Registry; Critical Care Registry, registers of Social Insurance Agency; National Diabetes Registry; and Cancer registry. The corresponding Norwegian registries will also be used.

Primary objectives will be evaluated after 5-years of follow-up.

Extended follow-ups of the patients beyond the 5-year study period are planned in order to evaluate long-term health effects and safety of SG vs GBP. These analyses are not covered in this analysis plan.

4. Study outcomes

A list of substantial adverse events in bariatric surgery (tabl 2) was predefined before study start. Possible occurrence of any of these will be identified in the CRF (SOReg) but additionally from the national quality health care registries (listed above) during 5-years of follow-up. A superiority of SG over GBP would require >35% fewer patients experiencing one or more substantial adverse event.

Time to occurrence of first substantial adverse event will be analysed as the co-primary outcome. Patients will be defined as either having or not having an event, and thus the number of events in the same patients will not be summarized.

For non-inferiority, weight loss difference over 5-years in patients operated with SG as compared to GBP will be evaluated with regards to the non-inferiority margin of 5%.

Table 1: Primary outcomes:

Outcome	Time frame	Explanation
Weight loss	At 5 years after surgery	Change in weight from baseline in % of total body weight
Substantial adverse events	Over 5 years after surgery.	Number of patients with any substantial adverse events defined according to table 2.

Table 2: Substantial adverse events: (events that may have a significant and/or longstanding effect on patients' health and/or everyday life).

**Substantial adverse event**	
Death	Death associated with bariatric surgery.
Intervention under general anesthesia	Complication according to Clavien-Dindo 3b or higher associated with the bariatric intervention (excluding gallstone interventions, preexisting hernia at bariatric surgery, and plastic surgery for removal of excess skin). A sub analysis of negative diagnostic laparoscopies is planned.
Admission to intensive care unit (ICU)	Admission to ICU due to complications from bariatric surgery.
Revision surgery	Change of surgical method; reversal or significant change of the original method. Revision surgery will be performed according to clinical practice. The indication for revision surgery (e.g. unsatisfactory weight loss or gastro-esophageal reflux) should be

	documented.
In-hospital care	Due to complications related to the surgery (excluding observation without diagnosis for less than 24 hours)
Frequent and severe food-related problems	Affecting everyday living for at least 3 months (such as hypoglycemia, pain, vomiting, nausea) in accordance with the "BEST/ SOReg template" available on the website.
Severe postprandial hypoglycemia	Hypoglycemic episodes associated with loss of consciousness/seizures or requiring drug treatment
Severe GERD	GERD symptoms that cannot be controlled with medication (e.g., volume reflux). Barrett's esophagus (with endoscopy performed within 2 years before surgery showing absence of Barrett).
Serious nutritional problems	Need for enteral (using a feeding tube to the gastrointestinal tract) or total/supplemental parenteral nutrition
Chronic abdominal pain	Pain (>6 months) requiring regular (> l time/week) medication with opioids (ATC code N02A, including tramadol and codeine) and/ or abdominal pain significantly affecting daily life (prohibiting normal work, leisure activities, etc.).
Substantial micronutrient deficiencies	Clinically relevant, symptomatic, vitamin and/or mineral deficiencies
Severe anemia	Hemoglobin <100 g/L in women and <110 g/L in men.
Other serious complication	Not pre-defined but, based on clinical data combined with national registry data, deemed to be a serious adverse event related to the surgery. If need be, these events can be assessed by a reference group.

Secondary outcomes:

Outcome	Time frame	Explanation
Arterial cardiovascular events	Baseline, 1, 2, and 5 years	Number of patients with Composite outcome including myocardial infarction, stroke and/or other occlusive arterial condition
Venous event	Baseline, 1, 2, and 5 years	Number of patients with composite outcome including deep vein thrombosis, pulmonary emboli, and/or other venous events
Diabetes requiring pharmacological treatment	Baseline, 1, 2, and 5 years	Development of diabetes measured as numbers of patients having a diabetes

		diagnosis with oral and/or injection treatment
Psychiatric morbidity	Baseline, 1, 2, and 5 years	Number of patients with pharmacological treatment for a psychiatric diagnosis
Hypertension	1, 2, and 5 years	Number of patients with pharmacological treatment for
Malignancy	Baseline, 1, 2, and 5 years	Number of patients diagnosed with any type of malignancy Data collected from Cancer registry
Dyslipidaemia	Baseline, 1, 2, and 5 years	Number of patients with oral lipid lowering treatment
Mineral nutritional status	2 and 5 years	Serum concentrations of iron depots, zinc, magnesium, selenium and copper, where available.
Vitamin nutritional status	2 and 5 years	Serum concentrations of vitamins (vitamin B12, vitamin D, vitamin A and thiamine), where available.
Serum concentrations of albumin	2 and 5 years	Serum albumin, where available.
Quality of Life	Baseline, 1, 2, and 5 years	EQ-5D, Obesity Problems (OP) and SF-36 scores
Alcohol consumption	1, 2, and 5 years	Alcohol consumption assessed by AUDIT, where available.
Gastro-esophageal reflux disease	1, 2, and 5 years	Gastro-esophageal reflux disease is defined as present if symptoms of GERD are reported with or without relief by PPI treatment.
Fractures	2 and 5 years	Diagnosis of any fracture collected from clinical data and national registry
Mortality and cause of death	5, 10, (20 and 30) years	National Cause of Death registry
Health care consumption and cost-effective analysis	5, 10, (20 and 30) years	Data from in-hospital registry (days in hospital), Outpatient registry (number of visits), and National drug registry (type

		and dose of drugs).<br><br>Formal cost-effective analysis with cost per quality-adjusted life-year and life-year.
Other co-morbidities	5, 10, (20 and 30) years	Development of co-morbidities from national registry data<br>(National stroke registry, National Cardiac registry, and National Diabetes Registry)
Peri-operative outcome	Within 30 days postop	length of stay (days), operating time (min), and complications surgical (minor/major) and medical.

5. Statistical methods

5.1 Sample size and power

The sample size calculation is based on evaluation of the primary objectives. For evaluation of superiority, it is assumed that 13% of patients who undergo GBP will have substantial adverse events (as defined in the Study outcomes) over 5 years. It is further assumed that this risk is 35% lower in patients in the SG group. Based on these assumptions, it is estimated that a total of 2100 patients (1050 in each group) will be required to detect a difference in time to substantial adverse event with >90% power at two-sided 2.5% significance level using Cox proportional hazards regression model.

Initially the power calculation was based on a SAE rate of 5% over 5 years, leading to the need to include 4000 patients (especially regarding subgroup analysis). Partly due to slower than expected inclusion rate, but mainly based on data suggesting more events of substantial adverse events than expected (i.e., 13%) the sample size was recalculated to n=2100 to obtain sufficient power.

This sample size will also have 90% power to evaluate non-inferiority of 5 % weight loss difference over 5 years between the two groups, assuming 15 kg standard deviation in weight loss over follow-up with two-sided 2.5% significance level.

Se appendix for an updated, post hoc, power-calculation before the closure of the trial inclusion.

5.2 Principles of analysis

The primary conclusions from this study will be based on analyses conducted under the principle of intention-to-treat. Thus, all randomised patients, with exception to those who withdraw their consent, will be included in the analyses. In the analysis, it is further assumed that all randomised patients receive the treatment they were randomised to.

Those patients who are randomised but withdraw their consent to use their data will not be included in any analyses. Only the information that they were enrolled into the study and withdrew their consent, and the original treatment group to which they were allocated, will be reported.

Data from all operating clinics (centres) will be pooled in primary analyses. The study will be conducted under a common protocol for each participating clinic with the intention to pool the data for analyses.

In addition to the primary analysis using intention-to-treat population, exploratory analyses using per-protocol (as treated) populations will be conducted for all outcome variables.

For primary outcomes, two-sided 2.5% significance level will be used to identify statistically significant results. The corresponding confidence intervals to be reported will be 97.5% confidence intervals. The use of 2.5% significance is due to two primary endpoints.

There will not be any formal interim analyses on primary outcomes. The safety and Data Monitoring Committee has a responsibility to monitor adverse events and complications during the study follow-up period.

5.3 Statistical analyses

Summary tables (descriptive statistics and frequency tables) will be provided for all baseline variables, outcome variables, and safety variables, as appropriate. Continuous variables will be summarized with descriptive statistics (n, mean, standard deviation, range, and median). Frequency counts and percentage of subjects within each category will be provided for categorical data.

Incidence of substantial adverse events will be analysed with time-to-event models. Time to first substantial adverse event (as defined in chapter 4 Study outcomes) from primary surgery will be recorded. Kaplan-Meier estimates of cumulative incidence of adverse events will be calculated in both treatment groups. Treatment effect will be calculated as a hazard ratio, together with corresponding confidence interval and p-value for the test of equal hazard ratio, from Cox proportional hazard regression model. The proportionality assumption of the Cox models will be evaluated graphically using "log-log" plots.

The primary conclusions will be based on a model which includes study centre as a covariate together with indicator for treatment. Explorative analyses will be conducted using sex, age, body mass index, smoking and diabetes at baseline as covariates.

For non-inferiority, the difference in weight loss between treatment groups and its confidence interval will be calculated and evaluated against the non-inferiority margin of 5%. The weight loss difference at 5-year follow-up will be estimated with a multilevel mixed-effect regression models utilizing body weight measurements at all available time points. This model takes into account the repeated measurements nested within persons over time. All randomized patients except those who have withdrawn their consent will be included in this analysis, independent of availability of body weight data during the follow-up. The primary conclusions will be based on a model which includes study center in addition to indicator for treatment.

For secondary outcomes, incidence of comorbidities during the follow-up will be evaluated with time-to-event models, and for continuous variables mixed-effect regression models will be used to evaluate differences between the treatment groups.

A priori defined subgroup analyses will be conducted by sex, age, diabetes state and baseline BMI for all outcomes. Age will be categorized in three groups (18-29; 30-45; > 45 years), and BMI as in two groups (<42 and >= 42kg/m^2).

5.4 Study follow-up and missing data

For evaluation of incidence of severe adverse events, it is expected that the coverage will be close to 100% as these are to be collected through national register linkage. Patients who withdraw consent will be censored right after withdrawal in time-to-event models. Patients who emigrate from Sweden or die before the end of 5-year follow-up will be censored at the time of emigration and date of death, respectively. All other patients not experiencing substantial adverse events during the study follow-up will be censored at the date of individual 5-year follow-up. The primary conclusions of the study will be based on analyses conducted on all randomized patients (with exception of those who withdraw their consent) under the principle of intention-to-treat.

For evaluation of non-inferiority, a sensitivity analysis will be conducted using both multiple imputation of missing body weight data, as well as last observation carried forward (LOCF) imputation. The results of these analyses will be reported together with results of the primary analysis.

5.5 Timing of analyses

Participant recruitment is planned to be completed by June 2019. Five-year follow-up will be completed for all participants during summer of 2024. Data-preparation will be completed by December 2024. Analyses and the results on primary outcomes will be reported during year 2025.

Appendix:

The unforeseen global Covid-19 pandemic resulted in that almost all elective benign surgery in Scandinavia was cancelled from March 2020. Thus, the pandemic had severe consequences on the recruitment to the BEST trial during 2020-2021.

During autumn of 2021 the BEST steering committee decided to perform an additional analysis of the power for primary endpoints.

Additional information (Courcoulas et al, JAMA Surg 2020 March; Howard et al, JAMA Surg 2021 Dec) revealed that the risk of any of the predefined substantial adverse events after bariatric surgery is higher than previously anticipated in the revised power calculation, i.e. >25% instead of 13%. These figures were confirmed in an analysis of real-world data from the bariatric national quality register SOReg in Sweden which registered all patients undergoing sleeve or bypass in Sweden since 2007.

An independent statistician performed the analysis based on information above, but also on 2-year data in BEST. In conclusion, it was stated:

Two post hoc power analyses were conducted based on the data from February 2022:

*1. Weight reduction. In the protocol the following is stated "This sample size will also have >95% power to evaluate non-inferiority of 5% weight loss difference over 5 years between the two groups, assuming 15 kg standard deviation in weight loss over follow-up with two-sided 2.5% significance level.". The post hoc power calculation is based on the two-year follow up data where an average weight loss for all patients (both groups, N=1031) of 29.3 kg was attained with a Sd=21.6. Given a loss to follow up of 20% from 2 year follow up to the 5-year follow up the sample size is assumed to be (1031*0.80)/2=413 patients per group. With a non-inferiority level of 5 kg weight loss the power is found to be 90% if n=393 per group, and 95% if n=486 per group (https://www.sealedenvelope.com/power/continuous-noninferior/).*

*2. Substantial adverse events. This post hoc power calculation is for a superiority test where the rate is assumed to be 25% for the gastric bypass at 5 years and sleeve would have a 35% lower level, i e 25%*0.65=16.25%. Given a sample of n=413 per group the post hoc power will then be 87.5%.*

Taking information above into account and in the interest of not prolonging inclusion period unnecessarily the trial steering committee took a decision to stop inclusion in BEST during spring 2022 (final date 31st of March). At termination of inclusion the number of participants that had been included and operated in BEST were 1752. The trial DSMC reviewed and supported the decision before termination of recruitment to the BEST trial.

Statistical analysis plan for:

Perioperative analysis of BEST- Bypass Equipoise Sleeve Trial

Randomized controlled Scandinavian multicentre study comparing gastric bypass and sleeve gastrectomy

version 4; Date: September 22th 2022

Acronym: BEST

Clinical Trial Registration Number: NCT02767505

Principal Investigator: Torsten Olbers

Senior statistician: Markku Peltonen

Plan prepared by: Suzanne Hedberg and Markku Peltonen

Contents

1. Introduction 3
2. Study objectives 3
3. Study Design 3
 3.1 Overview 3
 3.2 Randomization 4
 3.3 Eligibility criteria 4
 3.4 Data collection and follow-up 4
4. Study outcomes 4
5. Statistical methods 5
 5.1 Sample size and power 5
 5.2 Principles of analysis 5
 5.3 Statistical analyses 6
 5.4 Study follow-up and missing data 6
 5.5 Timing of analyses 6

1. Introduction

Gastric bypass (GBP) has been the standard surgical intervention to treat severe obesity in Sweden. Sleeve gastrectomy (SG) is a more recently introduced surgical technique, which has been used increasingly both in Sweden and internationally. Data from some smaller RCTs report no major differences between SG and GBP in terms of weight loss or resolution of comorbidities at short- and median-long follow up (Peterli, Salminen). However, the long-term effects of SG on body weight and health, as well as on complications and adverse events are less well described.

The current investigator-initiated study is a randomized, parallel group, Scandinavian multicentre study comparing long-term health effects and complications of GBP and SG. The study aims to evaluate a potential safety advantage of SG over GBP, and makes an efficacy comparison between the two treatments.

This plan is created to complement and expand upon the BEST: *Study Protocol*, *Statistical Analysis Plan (SAP),* and Method article (DOI: 10.1016/j.cct.2019.07.001), in matters specifically attaining to the baseline and perioperative data analysis.

2. Study objectives

The overall objective of the Bypass Equipoise Sleeve Trial is to evaluate the safety and effectiveness of SG compared to GBP in obese patients eligible for bariatric surgery.

The objective of the baseline and perioperative analysis is to present baseline and surgical data and to evaluate complications, re-operations, morbidity and mortality in the perioperative timeframe.

Predefined subgroup analyses will be done by sex, age, diabetes state and body mass index (BMI).

3. Study Design

3.1 Overview

This is a randomized, Scandinavian multi-centre trial comparing the two most abundant bariatric surgery techniques worldwide; laparoscopic gastric bypass (GBP) and laparoscopic sleeve gastrectomy (SG). Study design is parallel group study with 1:1 allocation between GBP and SG. The follow-up time for the primary objectives is 5-years.

The trial has been conducted at 23 bariatric surgery clinics in Sweden and Norway (Oct 6, 2015 – Mar 31, 2022), and includes 1752 obese patients approved for bariatric surgery, all of whom have been registered in SOReg.

3.2 Randomization

For detailed information on the randomization process please refer to *Statistical Analysis plan of BEST-Bypass Equipoise Sleeve Trial, version 5.*

3.3 Eligibility criteria

The target group is adult (>18 years) patients with severe obesity (BMI 35-50 kg/m^2) eligible and approved for bariatric surgery in Sweden. To be included in the study, patients need to fulfil all inclusion criteria to be eligible for operation using both techniques. Detailed inclusion and exclusion criteria are provided in the study protocol.

As per the study protocol patients are to be excluded from analysis, even though randomized, if a hiatal hernia of more than 4 cm is found at the start of surgery.

3.4 Data collection and follow-up

Baseline data regarding demographics and health parameters will be collected by the operating clinics using standardized questionnaires and measurements. Baseline data was collected at a presurgical visit approx. 2-8 weeks before surgery.

The data collection is facilitated using a central computerized system within the Scandinavian Obesity surgery Registry - SOReg.

Clinical health examination of the patients will be conducted baseline, 6 weeks (collecting 30-day data), 1 year, 2 years, and 5 years after surgery. The analyses described in this perioperative analysis plan refer to the timepoints of baseline, surgery and 30-day follow-up.

The perioperative analysis utilizes data collected at baseline, at surgery and at 30-day follow-up.

4. Study outcomes

The perioperative analyses refer to evaluation of baseline data, surgical data and substansive adverse events within 6 weeks of the surgery. Baseline data is description of the clinical characteristics of the study participants, se table 1. Evaluation of surgical data refers to describtion on how surgery was performed and deviations from protocol (table 2). Evaluation of substantial adverse events at 30 days, as listed in Table 3, comparison of GBP and SG.

118 Table 1: Baseline data

**Baseline data**	Collected at preoperative visit 2-8 weeks before surgery
Sex	Defined as male or female
Age at surgery	In years
BMI	BMI calculated using weight just prior to starting preoperative VLCD-diet, if such diet is utilized at hospital.
Current medical treatment	Y/N
Sleep apnea	Diagnosis of sleep apnea, with or without use of CPAP
Medical treatment for hypertension	Y/N
Medical treatment for diabetes	Y/N (all types of diabetes medication, including insulin)
Medical treatment for dyslipidemia	Y/N
PPI treatment for dyspepsia?	Y/N
Depression	Current diagnosis and/or medication
Experiencing muscle/skeletal pain	Y/N
Prior Deep Vein Thrombosis or Pulmonary Embolism	Y/N
Smoking	Non-smoker, Current smoker, Temporary smoking cessation in conjunction with current surgery, Former smoker

119

120 Table 2: Surgical Data

**Surgical Data**	
Operating hospital	
Surgical access	Laparoscopic/Open
Conversion from laparoscopic to open surgery?	Conversion because of: large liver; bleeding; equipment problems; adhesions; port placement problems; short

	mesentery; for other reasons
Surgical Method	Gastric Bypass (GBP) or Sleeve gastrectomy (SG)
Length of cartridges in GJ in mm (GBP)	
How many cartridges were used for the JJ (GBP)	
Distance Treitz-JJ (GBP)	Surgeons' estimation of length in cm
Distance GJ-JJ (GBP)	Surgeons' estimation of length in cm
How long division of the mesentery has been done in construction of the Roux-limb (GBP)	Surgeons' estimation of length in cm
Closure of Petersen's space (GBP)	
Closure of mesenteric defect at the JJ (GBP)	
Bougie diameter (SG)	In Chariére
How long from pylorus did the resection start (SG)	Surgeons' estimation of length in cm
Has the staple line been reenforced (SG)	Y/N, (How was the staple line reenforced (SG) and Which suture material was used for reinforcement? (SG))
How far from the angle of His does the resection end? (SG)	Surgeons' estimation of length in cm
Antibiotic prophylaxis?	Was antibiotic prophylaxis used? Y/N
Thrombosis prophylaxis?	No, Yes-LMW heparin low dose; Yes, LMW heparin high dose; Yes-other
Identification of hiatal hernia in	Y/N

surgery	
Axial length of hiatal hernia	Surgeons' estimation of length in cm
Other concurrent surgery?	Gynecological surgery; Cholecystectomy; Incisional or umbilical hernia; Splenectomy; Adhesiolysis for more than 10min; Cruroplasty (and Type of cruroplasty); Other concurrent surgery
Complications during surgery	Splenic injury; Unintentional bowel injury; Other complication (What other complication)
Operating time (min)	
Bleeding	0-99 ml, 100-499ml, 500-1500ml, >1500ml
Nr of days in hospital	(Registered at 6 weeks, but pertaining to surgical stay)

Table 3: Substantial adverse events: (events that may have a significant and/or longstanding effect on patients' health and/or everyday life).

**Substantial adverse event**	
Death	Death associated with bariatric surgery.
Intervention under general anesthesia	Complication according to Clavien-Dindo 3b or higher associated with the bariatric intervention (excluding gallstone interventions, preexisting hernia at bariatric surgery).
Admission to intensive care unit (ICU)	Admission to ICU due to complications from bariatric surgery. (Clavien-Dindo 4 or higer)
Revision surgery	Change of surgical method; reversal or significant change of the original method. Revision surgery will be performed according to clinical practice. The indication for revision surgery (e.g. unsatisfactory weight loss or gastro-esophageal reflux) should be documented.
In-hospital care	Due to complications related to the surgery (excluding observation without diagnosis for less than 24 hours)
Other serious complication	Not pre-defined but, based on clinical data combined with national registry data, deemed to be a serious adverse event related to the surgery. If need be these events can be assessed by a reference group.

5. Statistical methods

5.1 Sample size and power

For detailed information on sample-size calculation and power, please refer to *Statistical Analysis plan of BEST-Bypass Equipoise Sleeve Trial, version 5.* The formal power calculation of the study is based on the primary end-point of the study. There are no formal power calculations for the perioperative analyses described here, and they are regarded as exploratory.

5.2 Principles of analysis

Those patients who are randomised but withdraw their consent to use their data will not be included in any analyses. Only the information that they were enrolled into the study and withdrew their consent, and the original treatment group to which they were allocated, will be reported.

Data from all operating clinics (centres) will be pooled in primary analyses. The study will be conducted under a common protocol for each participating clinic with the intention to pool the data for analyses.

The analysis of perioperative data will be conducted per protocol.

5.3 Statistical analyses

Summary tables (descriptive statistics and frequency tables) will be provided for all baseline variables, outcome variables, and safety variables, as appropriate. Continuous variables will be summarized with descriptive statistics (n, mean, standard deviation, range, and median). Frequency counts and percentage of subjects within each category will be provided for categorical data.

Statistical significance for the differences between the two treatment groups will be evaluated with Fisher's exact test for categorical variables, and t-test for continuous variables.

For surgical and adverse event variables, a priori defined subgroup analyses will be conducted by sex, age, diabetes state and baseline BMI for all outcomes. Age will be categorized in three groups (18-29; 30-45; > 45 years), and BMI as in two groups (<42 and >= 42kg/m^2). Statistical significance of potential differences between the treatment groups and the subgroups will be evaluated with interaction analyses with logistic regression models for dichotomous variables and linear regression models for continuous variables.

5.4 Study follow-up and missing data

For evaluation of incidence of severe adverse events, it is expected that the coverage will be close to 100% as there is a very high adherents to 6-week registration. For the incidence of death, the

coverage is expected to be 100% as SOReg is routinely compared to the national death registry every other month.

5.5 Timing of analyses

Participant recruitment was completed on March 31st, 2022. Six-week follow-up was completed for all participants in May 2022. Data-preparation will be completed by September 2022. Analyses and the results on primary outcomes will be reported during spring 2023.

Version history:

version 4; Date: September 22th 2022: Tables of variables for baseline and surgical data added.

version 3; Date: September 20th 2022: Details regarding the statistical analyses added.

version 2; Date: September 15th 2022: Editing, removal of data preparation information.

version 1; Date: June 6th 2022: First draft.
