# Supplementary material for: Comparison of Sleeve Gastrectomy vs Roux-en-Y Gastric Bypass: A Randomized Clinical Trial
Source: JAMA Netw Open. 2024 Jan 30;7(1):e2353141. doi: 10.1001/jamanetworkopen.2023.53141 (PMC10828911; doi:10.1001/jamanetworkopen.2023.53141)
Supplement: Supplement 2. — eFigure 1. Reasons for Exclusion Among Eligible Patients Being Asked About Inclusion in the Randomization Module in SOReg eFigure 2. Distribution of Operating Time According to Procedure. Sleeve Gastrectomy (SG), Roux-en-Y Gastric Bypass (RYGB) eFigure 3. Reasons for Readmission for the 61 Patients Readmitted Within 30 Days eTable. Complications, Operation Time and In-Hospital Stay, Analyzed by Participating Hospitals, in Patients Randomized to Sleeve Gastrectomy and Roux-en-Y Gastric Bypass in the Bypass Equipoise Sleeve Trial (BEST) [file jamanetwopen-e2353141-s002.pdf]

Supplementary Online Content

Hedberg S, Thorell A, Österberg J, et al. Comparison of sleeve gastrectomy vs Roux-en-Y gastric bypass: a randomized clinical trial. *JAMA Netw Open*. 2024;7(1):e2353141. doi:10.1001/jamanetworkopen.2023.53141

eFigure 1. Reasons for Exclusion Among Eligible Patients Being Asked About Inclusion in the Randomization Module in SOReg
eFigure 2. Distribution of Operating Time According to Procedure. Sleeve Gastrectomy (SG), Roux-en-Y Gastric Bypass (RYGB)
eFigure 3. Reasons for Readmission for the 61 Patients Readmitted Within 30 Days
eTable. Complications, Operation Time and In-Hospital Stay, Analyzed by Participating Hospitals, in Patients Randomized to Sleeve Gastrectomy and Roux-en-Y Gastric Bypass in the Bypass Equipoise Sleeve Trial (BEST)

This supplementary material has been provided by the authors to give readers additional information about their work.

© 2024 Hedberg S et al. *JAMA Network Open.*

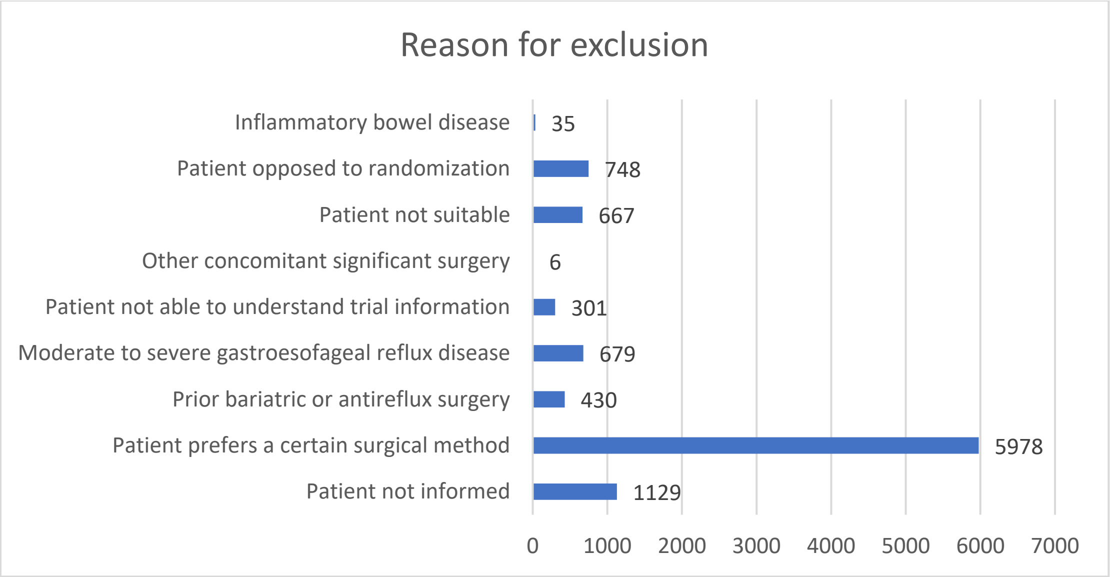

eFigure 1. Reasons for Exclusion Among Eligible Patients Being Asked About Inclusion in the Randomization Module in SOReg

Reason for exclusion was not recorded when using envelop randomization at Swedish hospitals before randomization module in SOReg (n=2454).

© 2024 Hedberg S et al. *JAMA Network Open.*

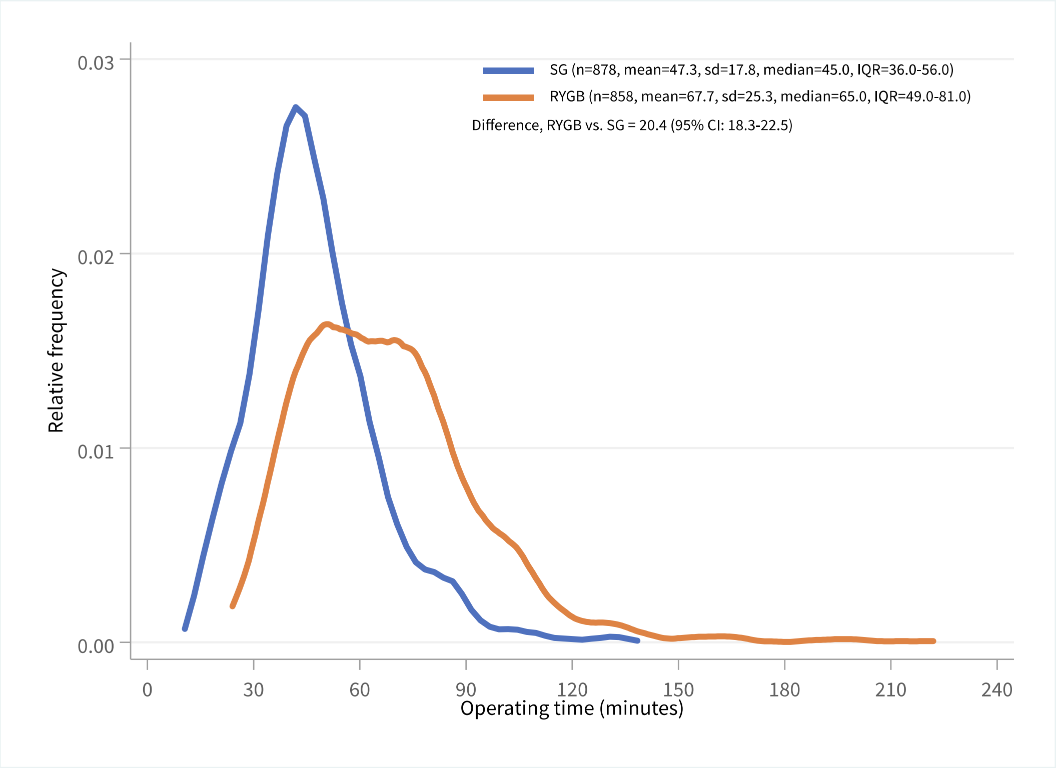

eFigure 2. Distribution of Operating Time According to Procedure. Sleeve Gastrectomy (SG), Roux-en-Y Gastric Bypass (RYGB)

© 2024 Hedberg S et al. *JAMA Network Open.*

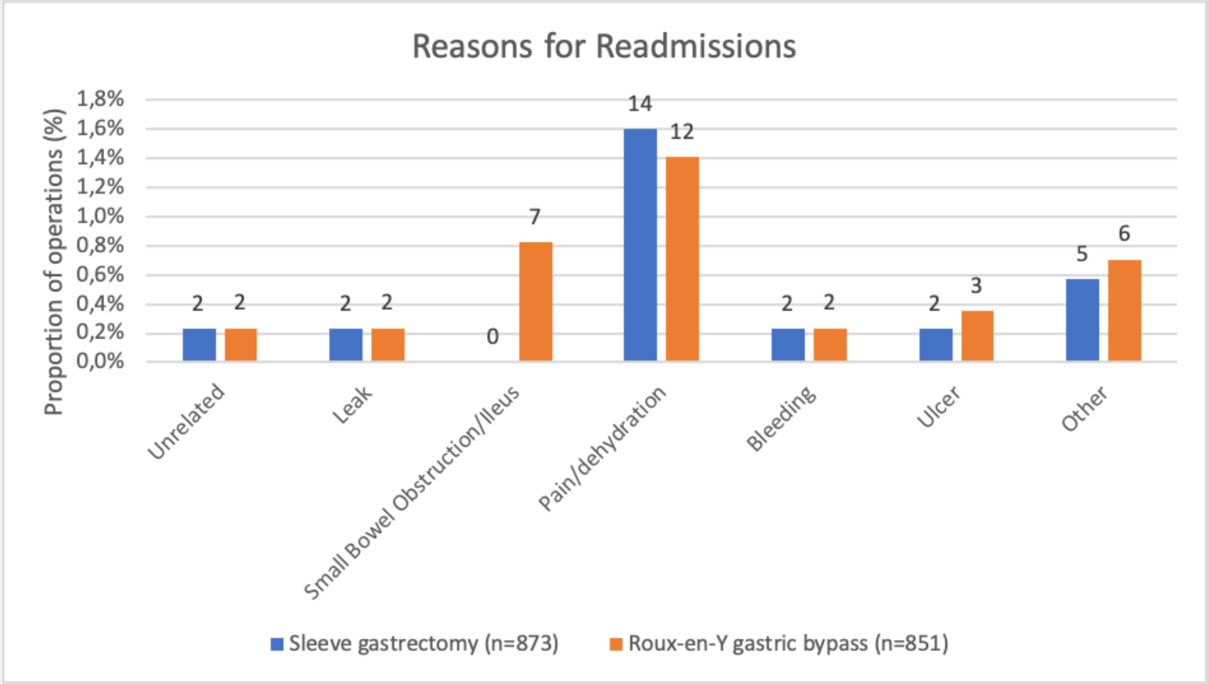

eFigure 3. Reasons for Readmission for the 61 Patients Readmitted Within 30 Days

Data labels refer to number of readmissions. Other refers to other diagnosis related to surgery, such as trocar site hernia, cutaneous abscess, pneumonia. Unrelated refers to admission to hospital within 30 days for reasons not pertaining to surgery, e.g., kidney stones.

© 2024 Hedberg S et al. *JAMA Network Open.*

eTable. Complications, Operation Time and In-Hospital Stay, Analyzed by Participating Hospitals, in Patients Randomized to Sleeve Gastrectomy and Roux-en-Y Gastric Bypass in the Bypass Equipoise Sleeve Trial (BEST)

Hospital, number	Total number	Any Adverse Event n (%)		Serious Adverse Event (Clavien-Dindo≥IIIb) n (%)		Surgery time (min) median (Range)		In-hospital (days) median (Range)	
		SG	RYGB	SG	RYGB	SG	RYGB	SG	RYGB
2	439	8(3.7)	15(6.8)	2(0.9)	7(3.2)	43(63)	52(98)	1(11)	1(7)
6	253	4(3.3)	8(6.1)	3(2.5)	3(2.3)	49(94)	79(158)	1(2)	1(42)
8	221	7(7.1)	11(8.9)	4(4.1)	4(3.3)	47(67)	77(123)	1(2)	1(27)
1	170	4(4.3)	6 (7.8)	1(1.1)	4(5.2)	62(96)	80(115)	1(10)	1(5)
10	68	1(2.4)	0(0.0)	0(0.0)	0(0.0)	48(73)	77(85)	1(8)	1(3)
Remaining[a]	67	1(2.5)	2(7.1)	0(0.0)	1(3.7)	42(77)	67(72)	2(2)	1(7)
5	62	1 (2.9)	0(0.0)	1(2.9)	0(0.0)	31(42)	47(45)	1(3)	1(0)
3+4[b]	59	2(5.9)	4(16.0)	0(0.0)	1(4.0)	54(102)	80(170)	2(46)	1(2)
12	56	2(5.9)	1(4.5)	1(2.9)	1(4.5)	20(28)	36(20)	1(4)	1(1)
20	48	0(0.0)	1(4.2)	0(0.0)	0(0.0)	20(18)	33(31)	1(0)	1(0)
21	47	3(13.0)	4(16.7)	0(0.0)	0(0.0)	28(30)	49(43)	1(3)	1(1)
11	42	1(5.0)	0(0.0)	1(5.0)	0(0.0)	45(47)	65(43)	1(1)	1(0)
7	41	0(0.0)	0(0.0)	0(0.0)	0(0.0)	30(32)	66(107)	1(1)	1(1)
16	38	0(0.0)	1(6.3)	0(0.0)	1(6.3)	55(93)	75(82)	2(2)	2(1)
15	37	1(5.3)	0(0.0)	0(0.0)	0(0.0)	39(41)	46(71)	1(2)	1(1)
22	35	2(11.8)	0(0.0)	1(5.9)	0(0.0)	41(40)	47(42)	1(3)	1(0)
14	28	3(20)	0(0.0)	1(6.7)	0(0.0)	51(43)	86(57)	1(13)	1(0)
18	27	0(0.0)	1(6.7)	0(0.0)	1(6.7)	29(21)	50(76)	1(0)	1(1)

[a] Hospitals (no. 9+13+17+19+23) including <25 (3–23) patients in BEST.
[b] Same hospital, in two geographic locations.

© 2024 Hedberg S et al. *JAMA Network Open.*
