# Supplementary material for: Comparison of Sleeve Gastrectomy vs Roux-en-Y Gastric Bypass: A Randomized Clinical Trial
Source: JAMA Netw Open. 2024 Jan 30;7(1):e2353141. doi: 10.1001/jamanetworkopen.2023.53141 (PMC10828911; doi:10.1001/jamanetworkopen.2023.53141)
Supplement: Supplement 3. — Nonauthor Collaborators [file jamanetwopen-e2353141-s003.pdf]

Supplemental Online Content: Nonauthor Collaborators
*First name, last name, and suffix (if applicable) are required and will appear in PubMed.

***Group Name(s): The BEST study group**							
***First Name and Middle Initial(s)**	***Last Name**	***Suffix (eg, Jr, III)**	Academic Degrees	Institution	Location (city, state/province, country)	Role or Contribution, eg, chair, principal investigator	Group (if more than 1 Group listed in the byline) and/or Subgroup (eg, Steering Committee)
Anna	Laurenius		RD, PhD	Department of Surgery, Department of Clinical Sciences, The Sahlgrenska Academy, University of Gothenburg, and Department of Gastroenterology and Hepatology, Sahlgrenska University Hospital	Gothenburg, Sweden	Member of BEST Steering Committee.	BEST study group
Jarl	Torgersson		MD, PhD	Department of Medicine, Department of Clinical and molecular medicine, The Sahlgrenska Academy, University of Gothenburg, and Department of Psychosis, Sahlgrenska University Hospital	Gotehnburg, Sweden	Member of BEST Steering Committee.	BEST study group
