# Supplementary material for: Comparison of Sleeve Gastrectomy vs Roux-en-Y Gastric Bypass: A Randomized Clinical Trial
Source: JAMA Netw Open. 2024 Jan 30;7(1):e2353141. doi: 10.1001/jamanetworkopen.2023.53141 (PMC10828911; doi:10.1001/jamanetworkopen.2023.53141)
Supplement: Supplement 4. — Data Sharing Statement [file jamanetwopen-e2353141-s004.pdf]

Data Sharing Statement

Hedberg. Comparison of Sleeve Gastrectomy vs Roux-en-Y Gastric Bypass. *JAMA Netw Open*. Published January 30, 2024. doi:10.1001/jamanetworkopen.2023.53141

Data
Data available: No

Additional Information
Explanation for why data not available: Data in this study can currently not be shared due to confidentiality under Swedish legislation and an ongoing follow up for assessment of the primary endpoint in BEST (5 years). Aggregated data from bariatric surgery in Sweden and Norway are available through the national quality registry SOReg (Scandinavian Obesity Surgery Registry, www.soreg.se and https://www.kvalitetsregistre.no/register/mage-og-tarm/norsk-kvalitetsregister-fedmekirurgi).
